# Beyond celiac disease: the potential role of gluten in Hashimoto’s thyroiditis

**DOI:** 10.3389/fendo.2026.1811207

**Published:** 2026-05-01

**Authors:** Xingye Fan, Xueyan Li, Yuan Fan, Yidan Fan

**Affiliations:** 1 The First School of Clinical Medicine, Yunnan University of Chinese Medicine, Kunming, Yunnan, China; 2 College of Traditional Chinese Medicine, Yunnan University of Chinese Medicine, Kunming, Yunnan, China; 3 Department of Endocrinology, The Second Affiliated Hospital of Yunnan University of Chinese Medicine, Kunming, Yunnan, China

**Keywords:** celiac disease, gluten, gluten-free diet, gut-thyroid axis, Hashimoto’s thyroiditis

## Abstract

Celiac disease (CD) and Hashimoto’s thyroiditis (HT) frequently coexist, suggesting that shared mechanisms of autoimmunity extend beyond the intestine. In CD, the pathogenic role of gluten is firmly established; in HT, however, gluten is better viewed as a candidate modifier rather than a proven universal trigger. This review synthesizes current evidence on how gluten may influence the CD-HT axis through gut dysbiosis, epithelial barrier dysfunction, immune cross-reactivity, and epigenetic regulation. We also examine the clinical evidence for gluten-free diet (GFD) use in three settings: classical CD, CD-HT comorbidity, and HT without confirmed CD. Current data support lifelong GFD in CD and suggest that patients with both CD and HT may gain indirect thyroid-related benefit as intestinal inflammation improves. By contrast, evidence remains insufficient to recommend routine gluten withdrawal for all patients with non-celiac HT. Long-term GFD also carries practical and nutritional burdens that require professional supervision. Overall, the most defensible clinical approach is targeted screening for CD or other gluten-related disorders in selected HT patients, followed by individualized dietary counseling rather than universal restriction. Future work should prioritize mechanistically informed, adequately powered randomized trials to identify which thyroid-autoimmune phenotypes, if any, are most likely to benefit from gluten exclusion.

## Highlights

- In CD, gluten is an established causal trigger and lifelong GFD remains standard treatment (4, 6, 85, 86).
- The CD-HT association likely reflects shared immune susceptibility and broader polyautoimmunity, not a single mechanism alone (18–20).
- Gut dysbiosis, increased permeability, and altered microbial metabolites provide a plausible gut-thyroid bridge (27, 30, 34, 35, 39).
- Direct evidence that gluten universally drives non-celiac HT remains insufficient (69, 72–75).
- Routine GFD for all HT patients is not currently evidence-based; individualized screening and counseling are preferable (69, 75, 81, 83).

## 1 Introduction

Gluten is a composite of storage proteins from wheat, barley, and rye, with gliadin and glutenin as its major fractions. Because gluten proteins are rich in proline and glutamine, they resist complete proteolytic digestion and generate immunogenic peptides that can persist in the intestinal lumen. In genetically susceptible individuals, these peptides are deamidated by tissue transglutaminase, presented mainly by HLA-DQ2 or HLA-DQ8, and drive both innate and adaptive immune activation. This pathogenic cascade is central to celiac disease, where gluten is an established environmental trigger rather than a hypothetical contributor (1–8).

HT is the most common autoimmune cause of hypothyroidism and results from loss of immune tolerance to thyroid antigens, particularly thyroid peroxidase and thyroglobulin. Its pathogenesis reflects the interaction of genetic susceptibility, environmental exposures, and immune dysregulation involving lymphocytic infiltration, cytokine imbalance, oxidative stress, and disordered Treg/Th17 homeostasis. Unlike CD, HT does not have a single universally accepted dietary trigger, which makes the proposed role of gluten more controversial and more clinically difficult to translate (9–15).

The association between CD and autoimmune thyroid disease is nevertheless clinically consistent. Patients with CD show increased rates of thyroid autoimmunity, and patients with HT have higher-than-expected rates of biopsy- or serology-confirmed CD. This overlap is better understood within a broader framework of polyautoimmunity: autoimmune thyroid disease is one of the most frequent components of overt or latent polyautoimmunity, while CD may also appear within autoimmune polyendocrine or polyautoimmune clusters. Framing the CD-HT link in this broader context helps explain why shared HLA background, barrier dysfunction, and common immune circuits may matter even when a direct gluten-thyroid causal pathway has not been definitively proven (16–20).

**Figure 1:**
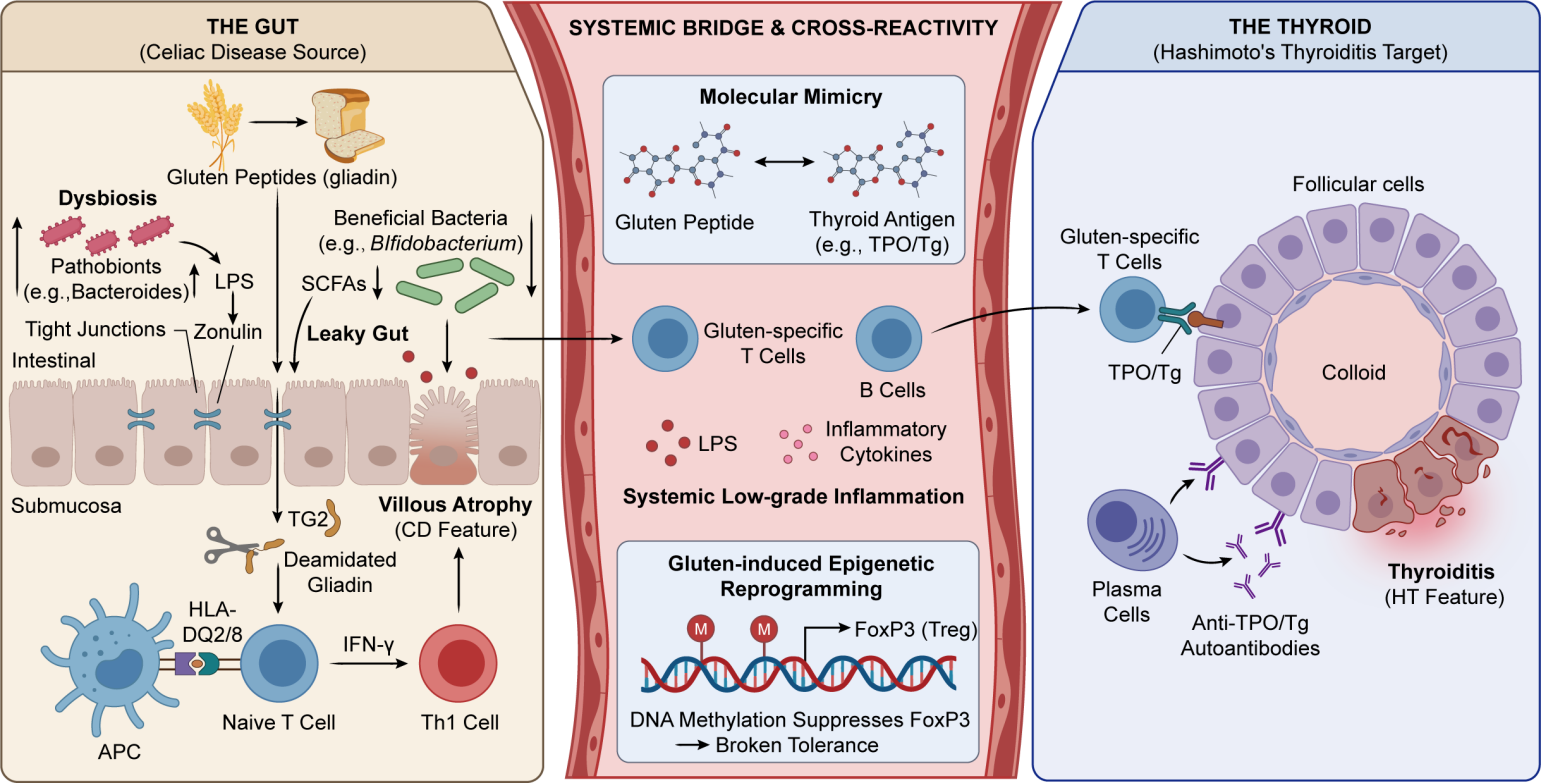
Integrated immunopathogenic mechanisms of the gluten-driven gut-thyroid axis. In genetically susceptible individuals, gluten exposure may promote dysbiosis, barrier dysfunction, mucosal immune activation, and systemic inflammatory spillover. These processes may converge with molecular mimicry and defective immune regulation to facilitate thyroid autoimmunity. The strongest evidence supports this pathway in CD, whereas its relevance to isolated HT is best interpreted as biologically plausible but not yet definitive.

Accordingly, this review focuses on the mechanisms most likely to connect gluten exposure with thyroid autoimmunity: gut dysbiosis and barrier failure, molecular mimicry and immune cross-reactivity, and epigenetic regulation of immune tolerance. We also critically reassess the therapeutic implications of a GFD, with particular attention to the difference between evidence-based treatment of CD and more selective, phenotype-driven use of gluten restriction in HT. The integrated immunopathogenic framework linking gluten exposure, intestinal events, systemic immune activation, and thyroid autoimmunity is summarized in **Figure 1**.

## 2 Gluten-induced gut dysbiosis and disruption of the gut-thyroid axis

### 2.1 Gut microbiota and immune homeostasis

The intestinal microbiota is a major regulator of mucosal and systemic immunity. Microbial metabolites such as short-chain fatty acids, bile acid derivatives, and tryptophan catabolites shape epithelial integrity, Treg differentiation, IL-22 signaling, and inflammatory tone. These effects are especially relevant to autoimmunity because they influence barrier function and peripheral tolerance at the same time. In early life, this interaction may be particularly important: gut bacteria can increase serotonin availability in the neonatal gut, thereby promoting tolerance to dietary antigens and commensal microbes. This observation provides a mechanistic rationale for why microbiota-dependent signaling could influence later autoimmune susceptibility (21–26).

### 2.2 Shared microbiota features in CD and HT

Both CD and HT are associated with gut microbial alterations, although the patterns are not identical across studies. In HT, systematic reviews suggest disrupted microbial composition, reduced representation of protective taxa such as Bifidobacterium and Lactobacillus, and evidence of increased intestinal permeability. In CD, dysbiosis is more consistently linked to disease activity and may persist even after initiation of a GFD. Across both disorders, a recurring theme is loss of potentially beneficial commensals, enrichment of taxa linked to inflammation, and functional changes that favor impaired barrier integrity and aberrant immune activation (27–35).

### 2.3 How gluten may reshape the gut-thyroid axis

Gluten may influence this axis in at least three ways. First, incompletely digested gluten peptides can alter the luminal substrate landscape and thereby favor bacterial communities with different proteolytic capacity. Second, gluten-driven inflammation can impair epithelial tight junctions and amplify IL-15- and cytokine-rich mucosal stress responses, creating a milieu in which dysbiosis becomes self-reinforcing. Third, altered microbial metabolism may shift the balance away from SCFA-rich, tolerance-supporting conditions toward lipopolysaccharide-rich, low-grade inflammatory states. Through these combined effects, gluten exposure may extend its consequences beyond the intestine and facilitate systemic immune activation relevant to thyroid autoimmunity (3, 36–40).

### 2.4 Metabolites, barrier dysfunction, and systemic spillover

Reduced SCFA production and increased permeability provide a plausible bridge between gut events and extraintestinal autoimmunity. In CD, barrier disruption enables the passage of immunogenic peptides and microbial products, promoting villous injury and sustained mucosal inflammation. In HT, elevated zonulin, lipopolysaccharide exposure, and other markers of barrier dysfunction have been linked to broader immune activation. Although these findings do not prove that gluten alone drives thyroid disease, they support the concept that a dysbiotic, permeable gut may lower the threshold for thyroid-directed autoimmunity in susceptible hosts (30, 34–36, 39, 41, 42).

## 3 Immune cross-reactivity between gluten, HT, and CD

### 3.1 Molecular mimicry as a general autoimmune mechanism

Molecular mimicry describes the situation in which foreign antigens and host antigens share sufficient structural similarity to permit cross-recognition by adaptive immune cells or antibodies. This mechanism has been implicated across multiple autoimmune diseases and provides a conceptual framework for linking environmental exposures to organ-specific autoimmunity. In thyroid disease, mimicry between infectious or microbial proteins and thyroid antigens has long been discussed; in gut-centered disorders, commensal-derived mimotopes can also activate autoreactive lymphocytes. Together, these observations make cross-reactivity biologically plausible in the CD-HT axis, even if the exact dominant antigens remain uncertain (43–47).

### 3.2 Shared immune architecture in CD and HT

CD and HT share more than epidemiologic overlap. Both diseases arise on a background of defective immune tolerance, exaggerated effector-cell responses, and altered innate signaling. In CD, HLA-restricted presentation of deamidated gluten peptides drives pathogenic CD4+ T-cell responses. In HT, thyroid-directed immunity is sustained by autoreactive T and B cells, inflammatory cytokines, and impaired regulatory control. Abnormal Treg/Th17 balance and heightened Toll-like receptor signaling have been described in both conditions, suggesting that similar immune architectures may support disease persistence once tolerance has been breached (2, 11, 13, 48, 49). The key concepts discussed in the main text and their clinical implications are summarized in **Table 1**.

**Table 1:** Key concepts discussed in the main text and their clinical implications.

Domain	Main message	Clinical implication	Representative references
Disease causality	Gluten is a confirmed trigger in CD but only a candidate modifier in HT.	Do not extrapolate the dietary certainty of CD to all HT patients.	(4, 9, 18)
Gut-thyroid axis	Dysbiosis, increased permeability, and altered metabolites may connect intestinal inflammation with thyroid autoimmunity.	Prioritize mechanistic studies and phenotype-based screening in symptomatic HT patients.	(27, 30, 34, 35, 39)
Immune cross-reactivity	Molecular mimicry is plausible, but direct proof of routine gluten-thyroid cross-reactivity remains limited.	Avoid overstating causality when counseling patients.	(18, 43, 47)
Epigenetics	Epigenetic dysregulation is shared by CD and HT, but gluten-specific thyroid epigenetic effects remain preliminary.	Treat epigenetic findings as hypothesis-generating rather than practice-changing.	(59–63)
GFD in CD-HT comorbidity	Benefits are clear for intestinal disease and may secondarily improve thyroid-related measures in some patients.	Strongly support GFD when CD is confirmed.	(64, 67, 68, 70)
GFD in non-celiac HT	Clinical evidence is inconsistent and generally insufficient for routine recommendation.	Use individualized assessment instead of universal restriction.	(69, 72–75)
Long-term management	GFD can impose nutritional, financial, and psychosocial burdens.	Dietitian-led follow-up is important whenever gluten restriction is prescribed.	(77, 78, 80, 81)

### 3.3 How strong is the evidence for gluten-thyroid cross-reactivity?

The strongest evidence for gluten-specific pathogenicity remains within CD. By contrast, direct evidence that gluten-specific immune responses routinely target thyroid antigens in HT is still limited. The literature is more convincing when discussing plausibility than proof: shared inflammatory pathways, barrier failure, exposure to microbial adjuvants, and mimicry hypotheses all support a possible connection, but they do not establish that gluten is a universal driver of thyroid autoimmunity. This distinction is important clinically. It justifies continued mechanistic investigation, while cautioning against overinterpretation of associative or low-powered studies when making dietary recommendations for HT (2, 3, 18, 47, 48, 50, 51).

## 4 Epigenetic mechanisms underlying the gluten-driven pathogenesis of HT and CD

### 4.1 Epigenetic control of immune tolerance

Epigenetic regulation influences how immune cells acquire identity, maintain plasticity, and respond to inflammatory stimuli. DNA methylation, histone modification, chromatin accessibility, and non-coding RNAs all contribute to T-cell differentiation, B-cell activation, macrophage polarization, and trained immunity. Because autoimmune disease reflects a failure of appropriate immune restraint, epigenetic dysregulation is a plausible upstream contributor to both CD and HT (52–56).

### 4.2 Shared epigenetic abnormalities in HT and CD

Epigenetic abnormalities have been reported in both disorders. In HT, altered microRNA signaling, DNA methylation changes, and barrier-related gene regulation have been linked to thyroid-cell vulnerability and proinflammatory T-cell responses. In CD, epigenetic changes involving intestinal barrier genes, immune signaling pathways, and oxidative-stress responses have also been described. These findings do not imply identical epigenetic signatures across the two diseases, but they do support the broader idea that common layers of transcriptional dysregulation may predispose to parallel autoimmune phenomena in the gut and thyroid (57–61).

### 4.3 Gluten as a possible epigenetic modifier

Whether gluten directly imprints durable epigenetic programs relevant to HT remains unresolved, but several observations justify continued study. In CD, persistent expression of endogenous retrovirus-related regulators despite GFD suggests that some immune alterations may outlast overt gluten exposure. In HT, dietary intervention studies have reported changes in epigenetic markers linked to FOXP3 regulation, although these data remain preliminary and difficult to separate from broader dietary effects. At present, epigenetics should be viewed as a promising explanatory layer rather than as definitive proof that gluten directly programs thyroid autoimmunity (62, 63).

## 5 Evidence and controversies regarding the GFD in the management of HT-CD comorbidity

### 5.1 Established value in CD and possible benefit in CD-HT comorbidity

For CD, the evidence is clear: a strict lifelong GFD remains the cornerstone of therapy, improves symptoms, lowers serologic activity, and supports mucosal recovery. In patients who have both CD and HT, gluten withdrawal may also improve the inflammatory context in which thyroid autoimmunity operates. Some studies have reported reductions in thyroid antibody titers or modest improvement in thyroid-related indices after GFD, particularly in selected subgroups or in patients with concomitant gluten-related disorders. Even so, the thyroid-specific signal is less robust than the intestinal benefit, and the literature remains heterogeneous in sample size, design, and outcome definition (64–71).

### 5.2 Non-celiac HT: promising hypothesis, insufficient evidence

The key clinical controversy is whether patients with HT but without confirmed CD should routinely avoid gluten. Here the evidence is notably weaker. Small pilot studies and some meta-analytic summaries suggest possible reductions in antibody titers or small shifts in thyroid function, but these effects are inconsistent and may be driven by highly selected populations, concurrent dietary changes, or unrecognized gluten-related symptoms. More rigorous recent studies, including randomized or genetically informed analyses, do not support a universal recommendation for GFD in non-celiac HT. The most evidence-based position is therefore individualized rather than routine use (64, 69, 72–75).

### 5.3 Why interpretation remains difficult

Three issues complicate interpretation. First, many studies are underpowered and rely on surrogate endpoints rather than patient-centered outcomes. Second, the term gluten-related disorders is often used imprecisely, which can blur the distinction among CD, non-celiac gluten sensitivity, and nonspecific gastrointestinal symptoms. Third, dietary intervention studies often alter multiple nutritional variables simultaneously, making it difficult to isolate the independent effect of gluten withdrawal. These methodological limitations help explain why enthusiasm for mechanistic plausibility has outpaced the quality of clinical evidence (69, 72, 75, 76).

## 6 Challenges in long-term management and implementation of GFD in CD and HT

Even when a GFD is medically indicated, long-term implementation is demanding. Adherence is undermined by hidden gluten exposure, eating-out restrictions, social burden, cost, and the variable nutritional quality of commercial gluten-free products. These concerns are particularly relevant in patients with CD-HT comorbidity, because thyroid management also depends on stable micronutrient intake and overall dietary adequacy. Restrictive diets imposed without clear indication may therefore create more burden than benefit (77–82).

Accordingly, dietary management should be stratified. In confirmed CD, a GFD is essential and should be supported by structured nutritional follow-up. In HT without CD, screening for celiac serology or other clinically relevant gluten-related disorders is more defensible than empiric long-term restriction for all patients. Multidisciplinary care involving endocrinologists, gastroenterologists, and dietitians is especially valuable when symptoms are complex or when nutritional compromise is a concern (79–81, 83).

## 7 Conclusion and future perspectives

Gluten has a proven pathogenic role in CD, but its role in HT is more nuanced. The current literature supports a biologically plausible connection through gut dysbiosis, barrier dysfunction, immune cross-reactivity, and epigenetic regulation, yet it does not justify treating gluten as a universal driver of thyroid autoimmunity. The best-supported clinical use of a GFD remains in patients with confirmed CD. For HT, particularly non-celiac HT, the evidence favors selective screening and personalized counseling rather than blanket dietary restriction (4, 9, 18, 69, 75).

Future studies should prioritize well-powered randomized trials, better phenotyping of gluten-related subgroups, and integrated multi-omics approaches that can test whether gut, immune, and thyroid signals converge in clinically meaningful ways. Distinguishing true gluten-responsive thyroid autoimmunity from coincidental dietary effects will be essential for moving the field from speculation to precision management (61, 69, 75, 81, 83, 84).
